# Stepped treatment algorithm using budesonide-formoterol for chronic respiratory diseases: A single arm interventional study

**DOI:** 10.1371/journal.pone.0271178

**Published:** 2022-07-11

**Authors:** Wan-Chun Huang, Greg J. Fox, Ngoc Yen Pham, Thu Anh Nguyen, Van Giap Vu, Viet Nhung Nguyen, Stephen Jan, Joel Negin, Quy Chau Ngo, Guy B. Marks

**Affiliations:** 1 Woolcock Institute of Medical Research, Hanoi, Vietnam; 2 South Western Sydney Clinical School, University of New South Wales, Sydney, Australia; 3 Division of Thoracic Medicine, Department of Internal Medicine, Shuang Ho Hospital, Taipei Medical University, Taipei, Taiwan; 4 Faculty of Medicine and Health, University of Sydney, Sydney, Australia; 5 Respiratory Center, Bach Mai Hospital, Hanoi, Vietnam; 6 National Tuberculosis Control Program of Vietnam, Hanoi, Vietnam; 7 Health Economics and Process Evaluation Program, George Institute for Global Health, Sydney Australia; 8 School of Public Health, University of Sydney, Sydney, Australia; PLOS: Public Library of Science, UNITED KINGDOM

## Abstract

**Background:**

While the safety and efficacy of inhaled budesonide-formoterol, used as-needed for symptoms, has been established for patients with asthma, it has not been trialed in undifferentiated patients with chronic respiratory diseases. We aimed to assess the feasibility of a pragmatic intervention that entails a stepped algorithm using inhaled budesonide-formoterol (dry powder inhaler, 160μg/4.5μg per dose) for patients presenting with chronic respiratory diseases to three rural district hospitals in Hanoi, Vietnam.

**Methods:**

We recruited patients with evidence of airflow obstruction on spirometry and/or symptoms consistent with asthma. The algorithm consisted of three steps: 1. as-needed inhaled budesonide-formoterol for symptoms, 2. maintenance plus as-needed inhaled budesonide-formoterol, and 3. referral to a higher-level healthcare facility. All participants started at step 1, with escalation to the next step at review visits if there had been exacerbation(s) or inadequate symptom control. Patients were followed for 12 months.

**Results:**

Among 313 participants who started the treatment algorithm, 47.2% had ≥ 1 episode of acute respiratory symptoms requiring a visit to hospital or clinic and 35.4% were diagnosed with an exacerbation. Twelve months after enrolment, 50.7% still adhered to inhaled budesonide-formoterol at the recommended treatment step. The mean and median number of doses per day was 1.5 (standard deviation 1.2) doses and 1.3 (interquartile range 0.7–2.3) doses, respectively. The proportion of patients taking more than 800μg budesonide per day was 3.8%.

**Conclusion:**

This novel therapeutic algorithm is feasible for patients with chronic respiratory diseases in a rural setting in Vietnam. Further studies are required to establish the effectiveness, safety and cost-effectiveness of similar approaches in different settings.

**Trial registration:**

ACTRN12619000554167.

## Introduction

Chronic respiratory diseases (CRD), including chronic obstructive pulmonary disease (COPD) and asthma, poses an enormous burden to health systems worldwide [1]. COPD and asthma are obstructive lung diseases that share common characteristics, such as treatment with inhalers, chronic airway inflammation, and exacerbations that are recognised by acute worsening of pulmonary function and respiratory symptoms.

Despite available evidence-based guidelines and cost-effective interventions, gaps exist between these approaches and actual clinical practice. Observational studies from different healthcare settings showed a low level of adherence to treatment recommended by guidelines, and insufficient awareness among physicians of optimal patient management [2–6]. Poor adherence to inhalers among patients has also been observed in various settings [7, 8]. There are major barriers to treatment for CRD in low- and middle-income countries (LMICs), including lack of access to diagnostic tests, limited human resources, and unavailability of medications [9–11].

Novel and pragmatic approaches should be considered to improve the uptake of effective medications in resource-limited settings. Recently, randomised trials have shown that inhaled budesonide-formoterol (IBF) in a single device, used as-needed, was as effective as daily maintenance inhaled corticosteroids in preventing exacerbations for mild and moderate asthma [12–14]. It is unclear if a similar approach can be used to achieve disease control for patients with all forms of obstructive lung diseases, including both asthma and COPD.

The aim of the study was to assess the feasibility of a pragmatic intervention that entails a stepped therapeutic approach using IBF for patients with CRD presenting to local healthcare facilities. We set two objectives to assess the feasibility, one to evaluate the process using ‘cascade of care’ and the other to evaluate the outcome by determining the proportion of enrolled patients with at least one exacerbation during a 12-month follow-up period.

## Materials and methods

### Study design and setting

This single-arm interventional study was conducted in three rural district hospitals in Hanoi, Vietnam. In Vietnam, district hospitals deliver care to populations of around 100,000 people and their local communities [15].

### Training for healthcare workers

Before the enrolment, healthcare workers from the three facilities participated in a training programme for study implementation, including recruit and follow-up participants, undertake patient education, administer inhaled medicine and perform spirometry and fractional exhaled nitric oxide (FeNO) testing. The study started at each facility with an assessment period that lasted for a week. During this period, research staff attended the facility to supervise the healthcare workers and deliver in-service training.

### Screening for CRD

Patients aged ≥ 12 years who presented to the facility with at least one of cough, dyspnea, wheeze, or chest tightness and had a history of at least one prior episode of respiratory symptoms that had required attendance at a healthcare facility within the past two years were screened.

The screening procedure included spirometry and a respiratory symptom questionnaire (RSQ) [16]. We performed spirometry using handheld EasyOne® Air spirometer (ndd Medizintechnik) according to American Thoracic Society/European Respiratory Society guidelines [17]. Spirometry results with a quality of “A” to “C” were considered valid [18]. Research staff assessed the quality of spirometric recordings during the assessment period and at site visits every two weeks.

Airflow limitation was defined as a pre-bronchodilator FEV_1_/FVC ratio < 0.7 or a peak expiratory flow < 0.8 of predicted value, if a valid FEV_1_/FVC result was not achieved. The RSQ includes nine questions assessing symptoms related to asthma in the past four weeks. A score of ≥ 3/9 gives a specificity of more than 90% and a sensitivity of around 70–80% for identifying individuals with a history of asthma in the last year or bronchial hyperresponsiveness determined by provocation test [16]. A score of ≥ 3/9 on the RSQ was defined as probable asthma.

Patients referred for screening meeting all the following criteria were eligible for the therapeutic intervention, whether or not there was a prior diagnosis of asthma or COPD: (a) having either airflow limitation, probable asthma, or both, (b) an alternative diagnosis, such as tuberculosis or pneumonia, was considered by clinicians to be unlikely to explain the symptoms, and (c) intended to live in Hanoi for the next 12 months. We excluded those who were (a) unable to provide consent, (b) allergic to budesonide or formoterol, and (c) pregnant women. Enrolled patients had a complete blood count with white cell differential count and FeNO measured at baseline. FeNO levels were categorised as low (<25 parts per billion, ppb), intermediate (25–50 ppb), and high (>50 ppb) [19].

### Stepped treatment algorithm and clinical follow-up

Patients enrolled for treatment were advised to use IBF (dry powder inhaler, 160μg/4.5μg per dose) according to a stepped algorithm. At step 1, patients used the inhaler only as required for relieving symptoms. At step 2, patients used the inhaler two actuations twice daily and, in addition, as required for relief of symptoms. At step 3, patients were referred for assessment by a specialist at provincial-level facility. Clinic doctors were advised to refer patients if considered necessary, such as a severe exacerbation that required more intensive management than was available at the district level.

All participants received an information leaflet about CRD and a management plan at the time of enrolment.

Pharmacists in the district hospitals instructed participants how to use the inhaler when they dispensed the patient’s first device. Afterwards, pharmacists checked and, if necessary, corrected inhaler technique each time a participant returned to collect a new inhaler device.

Every patient started at step 1 of treatment. Participants were asked to return to the clinic four weeks later for assessment. After the 4-week visit, the treating doctors decided the schedule of further appointments based on their judgement.

At each follow-up assessment, the treating doctors evaluated inhaler use by the participants, including inhaler technique and doses used based upon device counters. The doctors also determined their symptoms and exacerbations. Treatment was escalated to a higher step if the participant demonstrated ongoing symptoms consistent with poor control, or had exacerbation(s), since the last visit, that was not due to poor adherence or incorrect inhaler technique. Poor symptom control was defined as a score of < 20 in a symptom questionnaire modified from the Asthma Control Test (replacing asthma with respiratory symptoms in the questionnaire) [20].

Treating doctors assessed the presence of adverse events by enquiry to participants. Any identified serious adverse event was reported to the principal investigator. All serious adverse event reports were submitted to the Human Research Ethics Committee at the University of Sydney.

### Study outcomes

There were two objectives to evaluate the feasibility of the intervention. The first objective of the study was to determine the proportion of participants who had at least one exacerbation during the follow-up period. We defined an exacerbation as acute worsening of respiratory symptoms that resulted in (a) a healthcare visit, (b) a diagnosis of exacerbation by a physician, or (c) a prescription of systemic corticosteroids. It is a common practice in Vietnam that patients visit private pharmacy to get medicines without a diagnosis by a physician. To take this into account in our analysis, we also showed the result of healthcare visits excluding private pharmacy. Second, we estimated the proportion of participants who completed each step in a pre-specified ‘cascade of care’ in the treatment of CRD. Steps in the cascade included: (1) patients who attended the health facilities, presenting with respiratory symptoms consistent with CRD, (2) patients who initiated diagnostic assessment, (3) patients who completed spirometry or peak expiratory flow test, (4) patients completing diagnostic assessment who were diagnosed with CRD, (5) patients with CRD who commenced IBF, according to the algorithm, (6) patients who attended re-assessment 4 weeks after initiation of therapy, (7) patients who were adherent to recommended treatment after treatment commencement up to 3, 6, 9, and 12 months.

Research staff enumerated consecutive patients visiting the health facility with respiratory symptoms during the assessment period. The average number of people presenting to the facility per day meeting eligibility criteria during the assessment period was then used to estimate the number of participants at the first step of the cascade.

We assessed treatment adherence (step 7 of the cascade) by comparing the treatment step recommended by doctor and participants’ actual use. A participant was defined as “use IBF as recommended” if the participant complied with doctor’s recommendation at that time point. “Adherent to IBF” was defined as using IBF, whatever doing frequency, at that time point and previous contacts. “Adherent to recommended treatment step” was defined as complying with doctor’s recommendation at that time point and previous contacts.

Research staff called the participants 4 weeks, 3 months, 6 months, 9 months, and 12 months following enrolment to collect data. A patient was considered lost to follow-up if two or more consecutive follow-ups were missed.

### Statistical methods

We described the characteristics of participants using frequencies, means with standard deviation, and medians with interquartile ranges. Post hoc comparative analyses were performed using multivariable logistic regression, with model covariates determined using a causal diagram (S1 Fig). Missing values for smoking status were imputed for the model analysis using Stochastic regression imputation with age, sex, and level of education as the observed data. The binary outcome was one or more exacerbations, defined as in study outcomes, versus no exacerbation during the 12-month follow-up. The effects of interest included baseline FeNO, baseline blood eosinophil count, and treatment adherence. Treatment adherence was scored on a scale of zero to four, with four indicating the use of IBF as recommended at all four phone calls (3, 6, 9, and 12 months) and zero none of these time points. Analyses were conducted using SAS® (v9.4, SAS Institute, Cary Corp. NC. USA).

### Sample size

The targeted sample size of participants enrolled for intervention was 300, with 100 from each hospital. As we expected 30% of patients to have at least one exacerbation within 12 months, this sample size allowed us to estimate the proportion of patients experiencing one or more exacerbations within a 95% confidence interval of ± 5.2%.

### Consent and ethical approval

Patients who were eligible for screening provided verbal consent before screening procedure. Patients who met the eligible criteria for intervention gave written informed consent.

The study was approved by the Human Research Ethics Committee at the University of Sydney (protocol number: 2018/769), and the Institutional Review Board of the Bach Mai Hospital, Hanoi, Vietnam (approval number: 3497/QD-BM). The study was retrospectively registered with the Australian New Zealand Clinical Trials Registry (ACTRN12619000554167).

## Results

**Fig 1:**
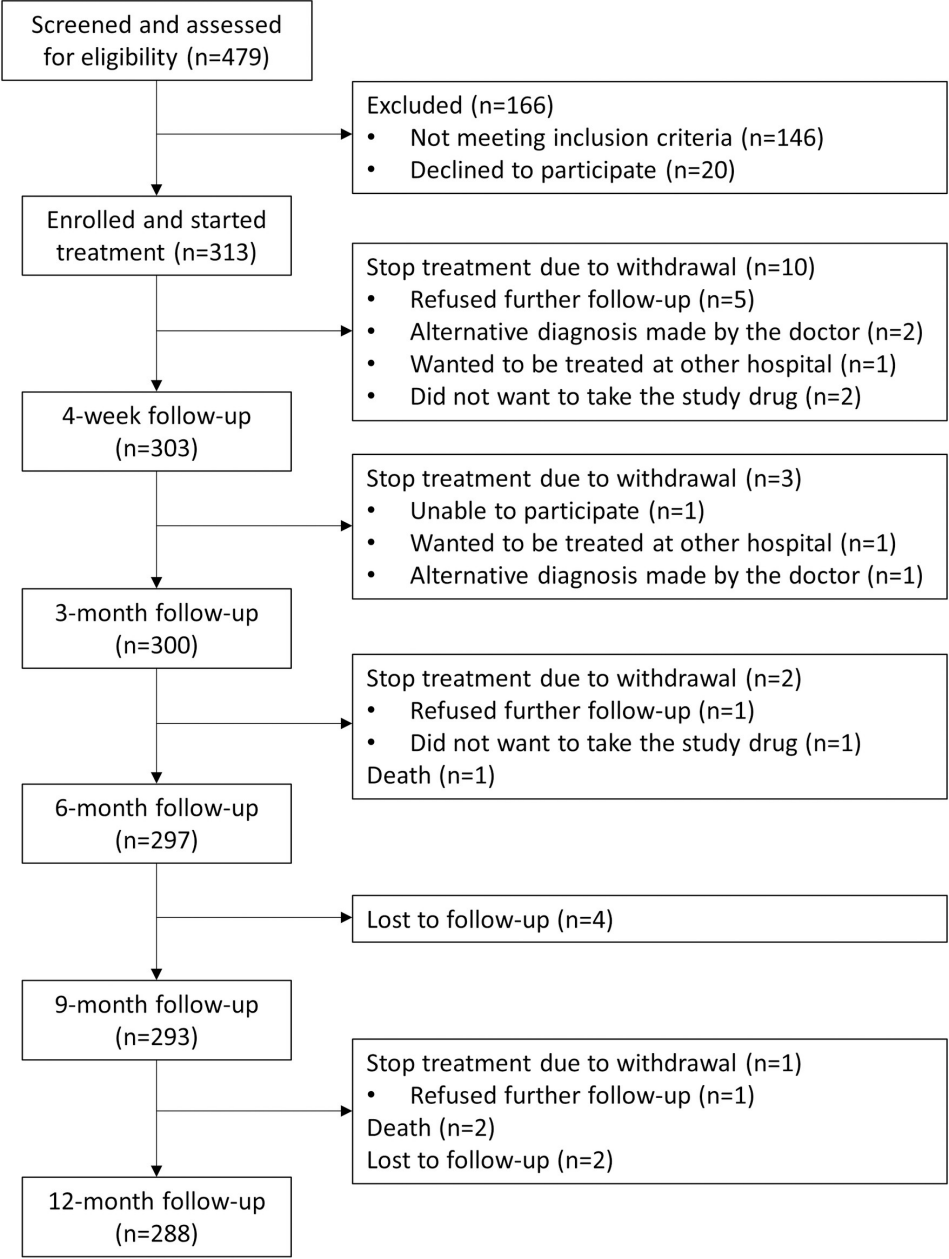
CONSORT diagram.

**Fig 2:**
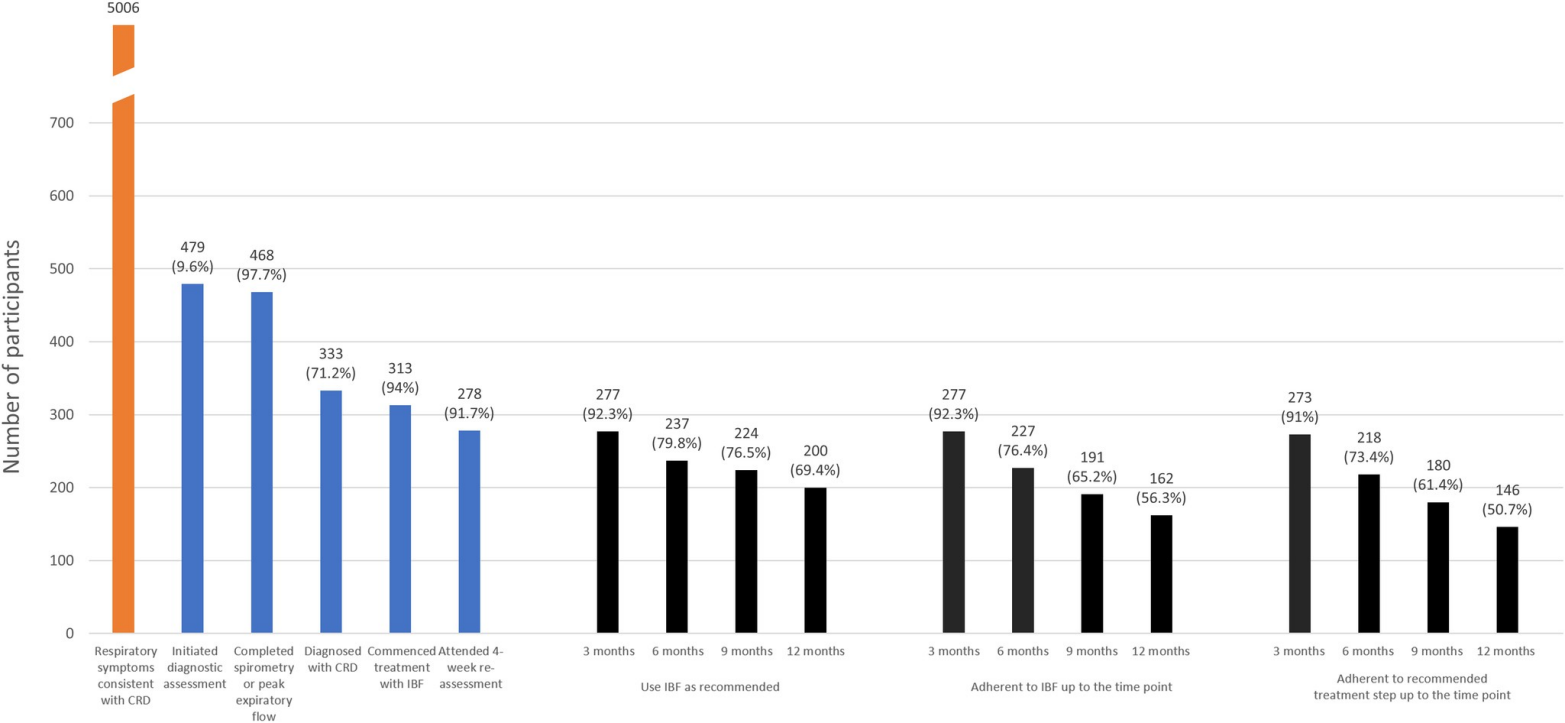
Proportion of patients completing each step of the cascade of intervention. CRD, chronic respiratory diseases; IBF, inhaled budesonide-formoterol.

Fig 1 shows the CONSORT diagram of the study. From March 2019 to July 2019, 479 patients were screened and initiated diagnostic assessment. Among them, 468 (97.7%) completed lung function and 391 (81.6%) had valid spirometry results (Fig 2). Among 333 (71.2%) patients diagnosed with CRD, 313 (94%) started the treatment algorithm. Based upon estimates obtained during the assessment period, the number of recruited patients comprised 9.6% of patients who visited the facilities with respiratory symptoms consistent with CRD.

The median age of the 313 patients was 65 years (interquartile range: 56–72 years, Table 1). Females accounted for 24.3% of the sample. Among 256 patients with an acceptable spirometry result, 230 (89.8%) had airflow limitation. Of 274 patients who had FeNO measured at baseline, 89 (32.5%) had an intermediate level and 60 (21.9%) had a high level.

**Table 1:** Baseline characteristics of study participants.

Characteristic	All participants
Total	313 (100)
Median age, years (IQR)	65 (56–72)
Female sex	76/313 (24.3)
Current smoking (n = 247)	86/247 (34.8)
Comorbidity	
Hypertension	66 (21.1)
Diabetes	18 (5.8)
Coronary artery disease	5 (1.6)
Heart failure	4 (1.3)
Gastrointestinal reflux disease	26 (8.3)
Respiratory symptom questionnaire ≥ 3	297/313 (94.9)
Baseline lung function*	
FEV_1_, litres (SD) (n = 256)	1.25 (0.6)
FVC, litres (SD) (n = 256)	2.2 (0.76)
FEV_1_/FVC (n = 256)	55.4 (12.1)
FEV_1_/FVC < 0.7 (n = 256)	230/256 (89.8)
Peak expiratory flow, %pred. (SD) (n = 50)	50.6 (23.1)
Peak expiratory flow %pred. < 0.8 (n = 50)	45/50 (90.0)
Eosinophil count, 10^9^/L (IQR) (n = 296)	0.27 (0.12–0.55)
FeNO, parts per billion (IQR) (n = 274)	26 (16–44)
Low level (< 25)	125 (45.6)
Intermediate level (25–50)	89 (32.5)
High level (> 50)	60 (21.9)
Highest level of education attained (n = 306)	
Less than primary education	33 (10.8)
Primary education	65 (21.2)
Secondary education	195 (63.7)
University degree, or equivalent, or higher	13 (4.3)

Data are median (IQR), n/N (%), or mean (SD).

FeNO, fractional exhaled nitric oxide; FEV1, forced expiratory volume in 1 second; FVC, forced vital capacity; IQR, interquartile range; SD, standard deviation

*Pre-bronchodilator.

Following enrolment, 278/303 (91.7%) participants attended the 4-week assessment (Fig 2). Twelve months after enrolment, 56.3% and 50.7% of participants were still adherent to IBF and to recommended treatment step, respectively.

**Fig 3:**
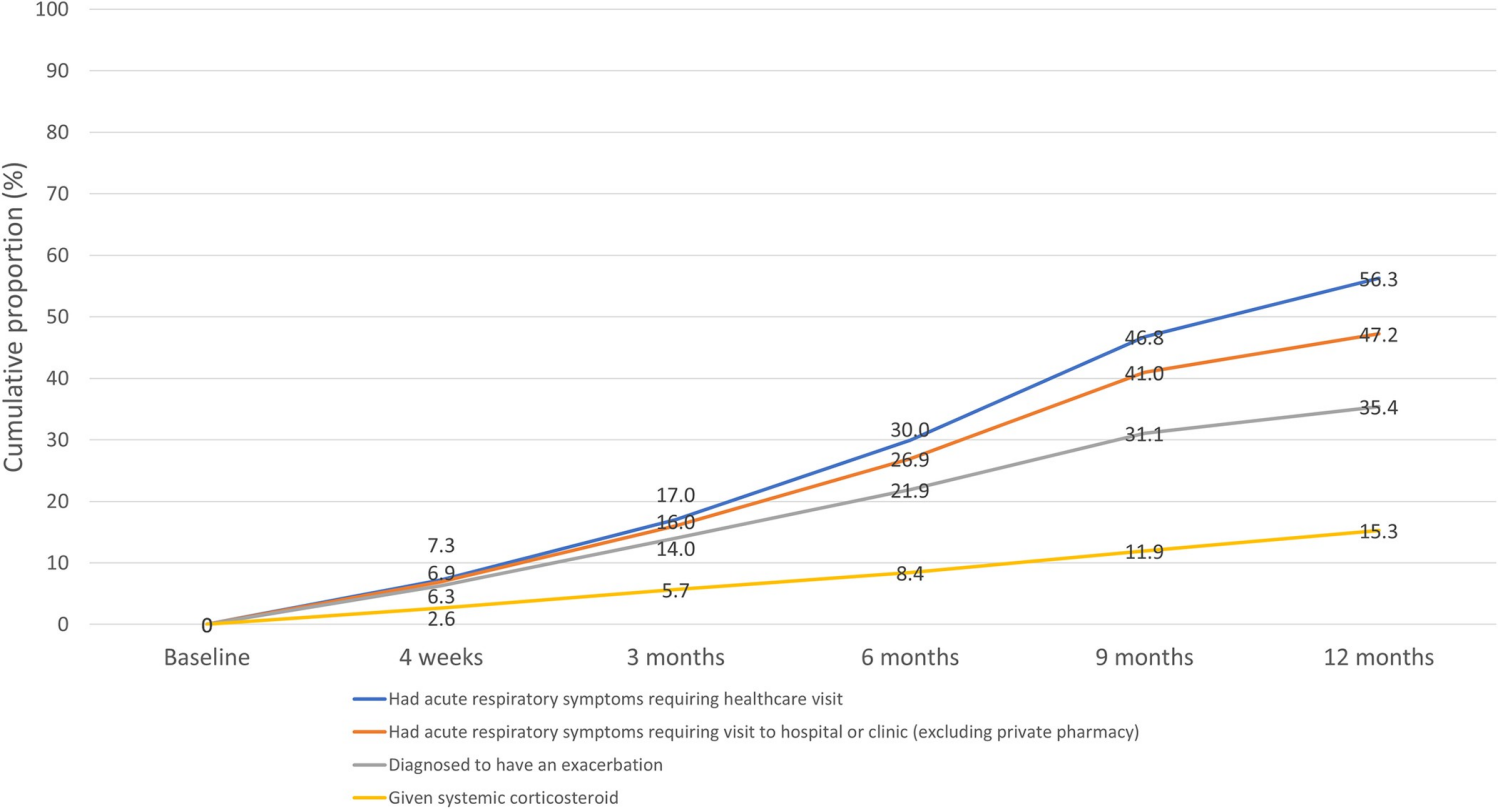
Proportion of participants with at least one exacerbation over study period.

The cumulative proportion of patients with an exacerbation, as defined, is shown in Fig 3. During the 12-month follow-up period, 56.3% of participants developed acute respiratory symptoms that required at least one visit to healthcare facility (47.2% if excluding private pharmacy visits). The proportion of participants diagnosed with one or more exacerbations and receiving systemic corticosteroids over the 12-month period was 35.4% and 15.3%, respectively.

**Fig 4:**
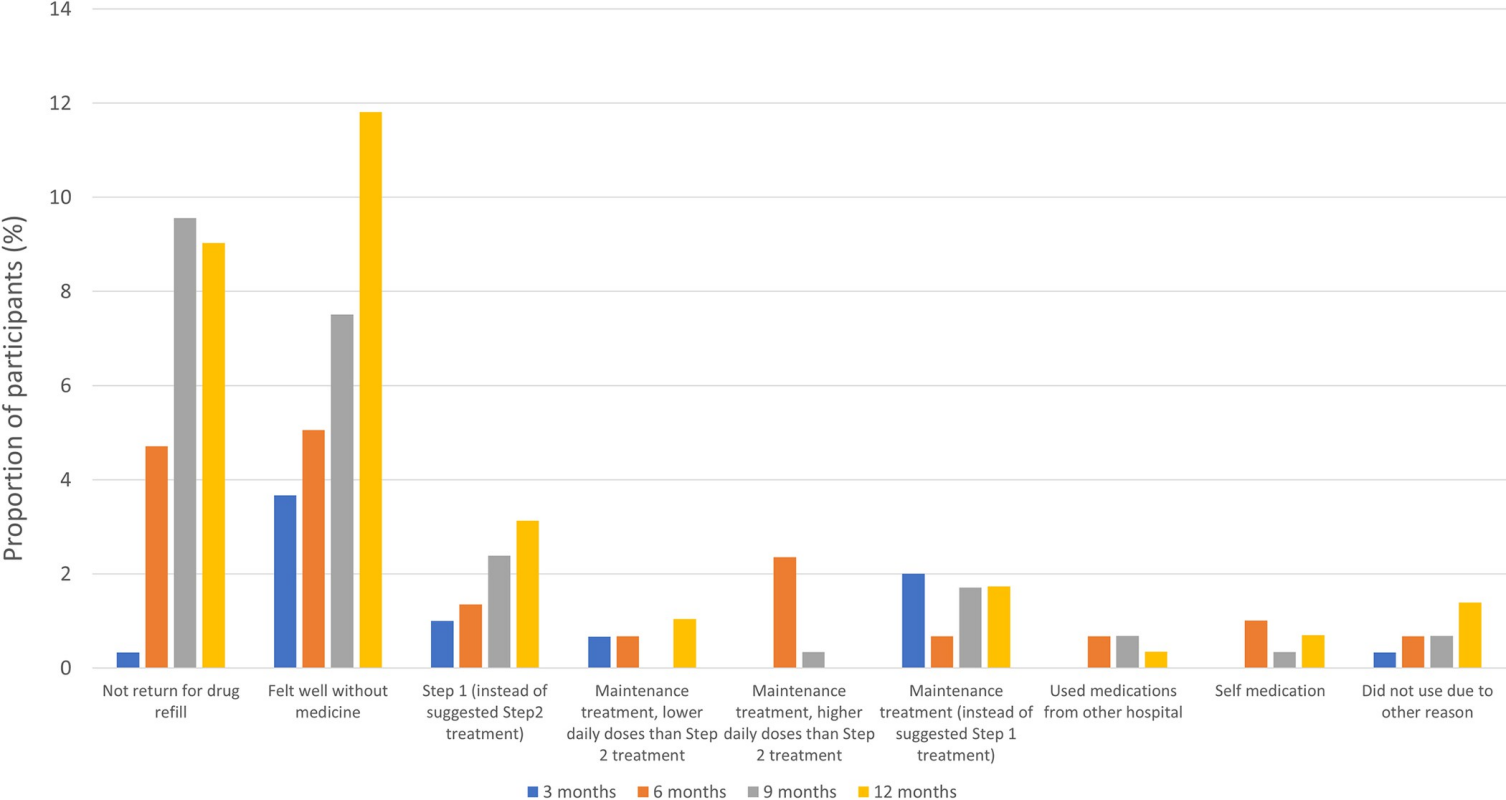
Patterns of nonadherence to treatment.

Fig 4 shows the prevalence of nonadherence to treatment. The proportion of patients who reported feeling well without using IBF and the proportion who reported using step1 treatment among those suggested to use step 2, both increased over time. Around 1% of patients continued maintenance treatment, but with a daily dose of less than that recommended for step 2 treatment.

The associations between exacerbations and effects of interest after adjustment are shown in Table 2. No association was found between exacerbation and the values of baseline FeNO or blood eosinophils. Treatment adherence was associated with a lower odds of visit to hospital or clinic (odds ratio = 0.712, 95% CI = 0.582–0.871, p = 0.001), a lower odds of diagnosis of exacerbation (odds ratio = 0.675, 95% CI = 0.536–0.85, p = 0.001), and a lower odds of being given systemic corticosteroids (odds ratio = 0.484, 95% CI = 0.307–0.763, p = 0.002).

**Table 2:** Logistic regression models of risk of developing exacerbations.

Effect of interest	Outcome	Adjusted odds ratio (95% CI)	Goodness-of-Fit (Hosmer-Lemeshow Test)	Covariates adjusted according to causal diagram
Baseline FeNO level* (intermediate vs low)	Visit to hospital or clinic	1.649 (0.932–2.916)	0.554	Baseline blood eosinophil count, smoking status
	Diagnosis of exacerbation by a physician	1.219 (0.673–2.210)	0.832	
	Given systemic corticosteroids	0.988 (0.453–2.152)	0.442	
Baseline FeNO level* (high vs low)	Visit to hospital or clinic	1.685 (0.875–3.242)	0.554	
	Diagnosis of exacerbation by a physician	1.627 (0.797–3.321)	0.832	
	Given systemic corticosteroids	2.268 (0.717–7.178)	0.442	
Baseline blood eosinophil count	Visit to hospital or clinic	1.173 (0.532–2.586)	0.554	Baselin FeNO level, smoking status
	Diagnosis of exacerbation by a physician	1.310 (0.560–3.065)	0.832	
	Given systemic corticosteroids	2.158 (0.585–7.958)	0.442	
Treatment adherence^**†**^	Visit to hospital or clinic	0.712^**§**^ (0.582–0.871)	0.210	Age, smoking status
	Diagnosis of exacerbation by a physician	0.675^**§**^ (0.536–0.850)	0.623	
	Given systemic corticosteroids	0.484^**§**^ (0.307–0.763)	0.820	

FeNO, fractional exhaled nitric oxide

*Low level, <25 ppb; intermediate level, 25–50 ppb; high level, >50 ppb

^**†**^Scored on a scale of zero to four, with four indicating the use of inhaled budesonide-formoterol as recommended at all four phone calls (3, 6, 9, and 12 months) and zero none of these time points

^**§**^Statistically significant.

Table 3 shows the average daily doses of IBF used by participants. Over the study period the mean daily number of doses ± standard deviation was 1.5 ± 1.2 doses and the median daily number of doses was 1.3 (interquartile range: 0.7 to 2.3) doses. Over the period from enrolment to the date of the last drug dispensing, these values were 2.3 doses ± 1.2 and 2.1 doses (interquartile range: 1.4–3.0 doses), respectively. The proportion of patients with an average daily budesonide dose exceeding 800μg was 3.8% over the entire study period and 8.8% over the period from enrolment to date of last drug dispensing.

**Table 3:** Average daily doses of budesonide-formoterol (N = 288).

Method of calculation*	Mean daily doses (SD)	Median daily doses (IQR)	Maximum average daily doses	Proportion with 5–8 daily doses^§^
**Total study period (365 days)**	1.5 (1.2)	1.3 (0.7–2.3)	5.3	3.8%
**Between first (date of enrolment) and last inhaler dispensing** ^**†**^	2.3 (1.2)	2.1 (1.4–3.0)	6.9	8.8%

*Two different methods used because doses used between the day the last inhaler dispensed and the last day of study were not known

^**†**^Excluding patients who had only one inhaler dispensed

^**§**^200 μg budesonide per dose.

Throughout the study period, four serious adverse events were reported, including three deaths and one intraventricular haemorrhage. The first patient continued Step 1 treatment until four months after the 4-week assessment. Five months after stop using inhaled budesonide-formoterol, the patient was hospitalised with the diagnosis of pneumonia and heart failure and died about 2 weeks later. The second patient experienced an episode of exacerbation about one month after the 4-week assessment and was treated as inpatient for one week. He then started Step 2 treatment. Three months later, he had another episode of exacerbation and was hospitalised for 2 weeks. The patient stopped using inhaled budesonide-formoterol but used another inhaler bought by family. The patient’s symptoms aggravated and the patient died about three weeks later after the second hospitalisation. The third patient discontinued inhaled budesonide-formoterol and self-treated with traditional herb about five months after the 4-week assessment. About four months later, the patient experienced chest pain and breathlessness and was diagnosed to have lung cancer. The patient died about a week later. The last patient was under stable condition until four months after enrolment when the treatment was escalated to Step 2 due to inadequate symptom control. Five months later, he fell at home and was diagnosed with intraventricular haemorrhage and hypertension at a central hospital. He was in good control with Step 2 treatment thereafter. None of the above adverse events was judged to be related to the study drug. One participant reported skin rash, which improved after discontinuing the study drug.

## Discussion

In this study we showed the feasibility of a novel and pragmatic therapeutic algorithm used for patients with CRD, including both asthma and COPD. More than half of participants complied with the recommended treatment up to 12 months after enrolment. Adherence to recommended treatment was associated with a lower risk of exacerbation. However, baseline FeNO and blood eosinophil counts were not related to the subsequent risk of having an exacerbation. Only a small number of patents required an average daily dose of budesonide of over 800 μg. The frequency of exacerbations among the participants and the safety profile of the therapeutic algorithm were within the expected range for patients with CRD [21–24].

Diagnostic assessment using spirometry is essential to identify patients with CRD. In our study, only 10% of patients with repeated respiratory symptoms underwent diagnostic assessment, suggesting a low rate of referral for spirometry in the facilities. Hence, many patients with CRD may have been missed. A recent cross-sectional survey showed more than 20% of patients with respiratory symptoms who attended district health facilities in Vietnam had either fixed or reversible airflow limitation [25], and would have potentially benefited from the treatment algorithm of this study. Other studies have also shown that misdiagnosis of COPD and asthma is common [26, 27]. Our study suggested that a portable spirometer can be effectively incorporated in clinical practice in a rural setting of LMICs to facilitate diagnosis and prompt proper treatment.

Poor adherence to inhaled medicine is a well-documented problem [28, 29]. A study of patients with COPD showed adherence could be as low as 13%, 12 months after starting maintenance treatment [30]. In our study, the proportion of participants remained adhering to suggested treatment was high compared to other published studies. Furthermore, the majority of patients who were nonadherent to treatment suggestions reported feeling well during follow-up, even with a self-directed dose reduction. Therefore, the observations support the feasibility of this treatment algorithm in this setting.

Increased FeNO and blood eosinophil counts at baseline were shown not associated with risk of exacerbations in our study, which was not consistent with evidence from studies in patients with COPD and asthma [31–33]. A recent trial assessing as-needed IBF in patients with mild asthma found a similar result [34]. From their analysis, benefits of as-needed IBF over as-needed salbutamol for preventing exacerbations were independent of baseline blood eosinophil count or FeNO. Given that the two biomarkers are known predictors of response to inhaled corticosteroids [35–37], it is plausible that exacerbations were prevented through a pathway involving formoterol among patients with a low level of type 2 inflammation [34]. More studies are required to show the relationship between these biomarkers and exacerbations among patients using as-needed IBF. Furthermore, even though our study did not seek to distinguish COPD and asthma, studies evaluating effects of as-needed IBF in patients with COPD may help expand our understanding of management of CRD.

Even though the estimation of exacerbations might be affected by concomitant cardiorespiratory diseases, or underestimated due to patients’ not reporting deterioration, we consider the proportion of exacerbation among participants within expected range. A prospective cohort in Uganda found 59.6% of patients with asthma experienced at least one exacerbation in a year [23]. Another study conducted in multiple Asia-Pacific countries reported 33.1% of patients with mild intermittent asthma and 58.6% of patients with severe persistent asthma required an emergency visit for respiratory condition during the previous year [22]. The PERCEIVE study showed 89% of people with COPD suffered from at least one episode of symptom flare-up within a year [24]. The exacerbation frequency in our population, alone with the adherence pattern, suggest the algorithm could be used in other similar settings. The effectiveness of the algorithm in reducing exacerbations and its safety are currently under investigation in Vietnam with a cluster randomised controlled trial (ACTRN12620000649910).

Smoking cessation is an essential part of care for patients with CRD. This study was part of a larger study that also encompassed interventions to assist smoking cessation taking place in the same district hospitals. Participants of the intervention of this paper were eligible for smoking cessation intervention if they met the inclusion criteria. The interventions were not exclusive and participants were not randomised to receive only one intervention. Among the participants of this intervention, 23 also received smoking cessation intervention. Of the 23 participants, 7 (30.4%) achieved self-reported abstinence for at least 30 days at 12 months and 4 (17.4%) achieved biochemical-verified abstinence at 12 months. The smoking cessation intervention and study results are described in another paper submitted for publication.

The study is novel in several ways. First, the treatment algorithm requires only one inhaled medicine. This could reduce the need for procuring multiple inhalers and prevent the problems of using various types of inhaler devices, such as poor technique and low adherence. Second, our population includes both patients with COPD and asthma. The algorithm does not require clinical staff be able to distinguish between the two entities, which is difficult in many clinical settings. Finally, the study was implemented in three rural district hospitals in Vietnam, indicating the potential utility of such algorithm in resource-limited areas and primary care.

This study has several limitations. First, the RSQ was originally designed for epidemiological studies [16] and its validity in directing therapy in clinical settings is not established. However, we found most patients enrolled had both airflow limitation and a high score in the questionnaire. Hence, it is unlikely that we inadvertently enrolled many patients without CRD. Second, data regarding exacerbation frequency was obtained from the participants and were not validated with medical records. Third, we did not assess the incidence of pneumonia and pulmonary tuberculosis, two possible adverse events of inhaled corticosteroids. Third, current tools to assess symptom control were designed for either COPD or asthma. The validity of using these tools for a population constituted by different forms of obstructive lung diseases remains to be explored. Finally, the lack of association between exacerbations and the two biomarkers could have been due to limited statistical power from relatively small sample size.

In conclusion, this novel therapeutic algorithm was feasible and tolerable for patients with CRD in a rural healthcare setting. Further studies are required to establish the safety, effectiveness and cost-effectiveness of similar approaches in a range of settings.

## Supporting information

S1 Checklist TREND statement checklist. (PDF)

S1 Fig Casual diagram (directed acyclic graph) for logistic regression models evaluating factors associated with risk of exacerbations. Figure produced from http://www.dagitty.net/dags.html.
(PDF)

S1 File Study protocol. The file contains the full protocol of a pilot study that assessed the feasibility of interventions to manage chronic respiratory disease and reduce smoking in Vietnam. This paper presents the findings of the chronic respiratory disease intervention described in the protocol.
(PDF)
