# Supplementary material for: Stepped treatment algorithm using budesonide-formoterol for chronic respiratory diseases: A single arm interventional study
Source: PLoS One. 2022 Jul 11;17(7):e0271178. doi: 10.1371/journal.pone.0271178 (PMC9273083; doi:10.1371/journal.pone.0271178)

S1 Fig. Causal diagram (directed acyclic graph) for logistic regression models evaluating factors associated with risk of exacerbations. Figure produced from http://www.dagitty.net/dags.html

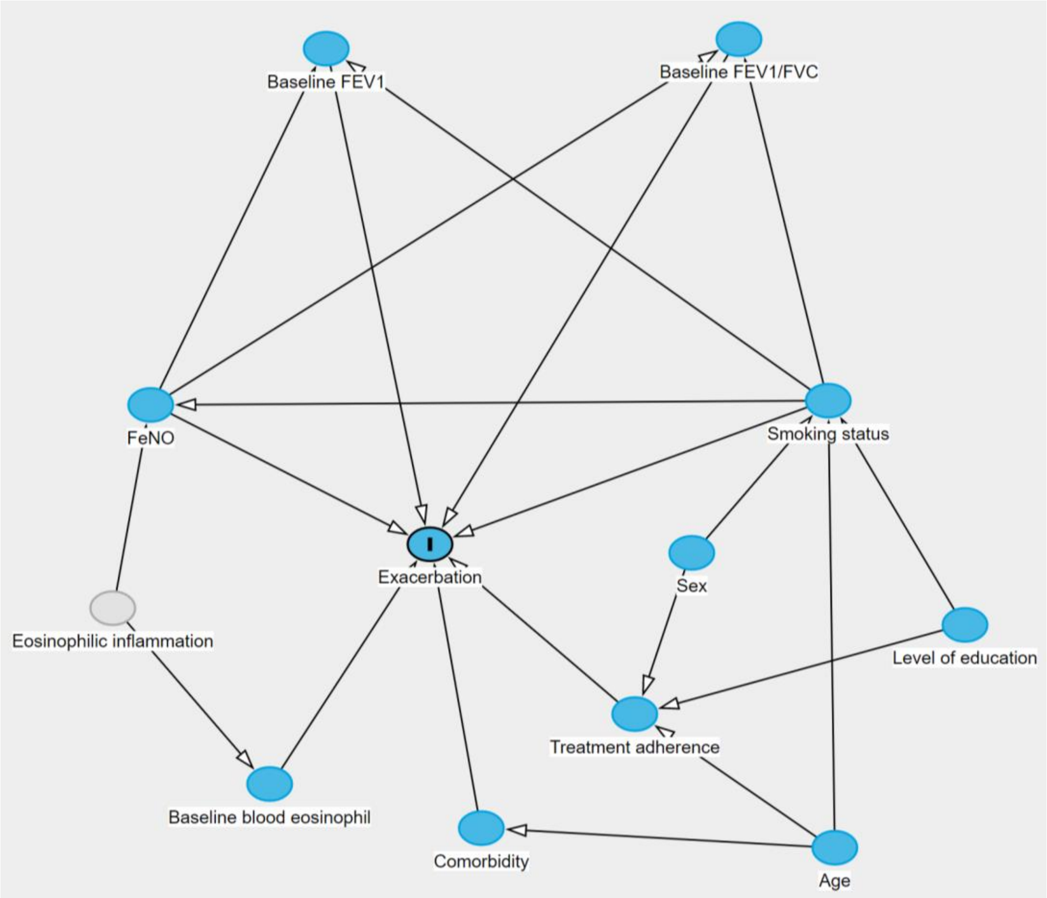

Supplement: S1 Fig — Figure produced from http://www.dagitty.net/dags.html. (PDF) [file pone.0271178.s002.pdf]
