# Supplementary material for: Stepped treatment algorithm using budesonide-formoterol for chronic respiratory diseases: A single arm interventional study
Source: PLoS One. 2022 Jul 11;17(7):e0271178. doi: 10.1371/journal.pone.0271178 (PMC9273083; doi:10.1371/journal.pone.0271178)
Supplement: S1 File — The file contains the full protocol of a pilot study that assessed the feasibility of interventions to manage chronic respiratory disease and reduce smoking in Vietnam. This paper presents the findings of the chronic respiratory disease intervention described in the protocol. (PDF) [file pone.0271178.s003.pdf]

A note to readers,

This document serves as a supporting information file to the *PLOS ONE* manuscript titled "Stepped treatment algorithm using budesonide-formoterol for chronic respiratory diseases: a single arm interventional study".

The study protocol that is found with the following pages is in its original language as it was submitted and approved by the Human Research Ethics Committee at the University of Sydney before the trial began.

This study was a pilot study to a cluster randomised controlled trial (ACTRN12620000649910). The feasibility of interventions to manage chronic respiratory disease and reduce smoking in Vietnam was assessed in this study. This *PLOS ONE* manuscript presents the findings of the chronic respiratory disease intervention described in the protocol. Results from the smoking cessation interventions are not included in this *PLOS ONE* manuscript.

WOOLCOCK INSTITUTE

OF MEDICAL RESEARCH

STUDY PROTOCOL

The VCAPS 3 Study

An integrated public health strategy to manage chronic respiratory disease and reduce smoking in three District Clinics in Vietnam: a prospective cohort study

---

January 2019 – December 2020

Version 12.3

---

1. SUMMARY

Title: An integrated public health strategy to manage chronic respiratory disease and reduce smoking in three district clinics in Vietnam: a prospective cohort study

Timeline: January 2019 – December 2020

Study investigators

Name	Title	Office
**Chief investigators**		
A/Prof Greg Fox	Principal Investigator<br>MBBS PhD	Woolcock Institute of Medical Research<br>University of Sydney
Prof Guy Marks	Investigator<br>MD PhD	Woolcock Institute of Medical Research<br>University of New South Wales
Prof Joel Negin	Investigator<br>PhD	Sydney School of Public Health, Faculty of Medicine and Health<br>University of Sydney
Prof Steven Jan	Investigator	George Institute for Global Health<br>University of New South Wales
**Associate Investigators**		
Dr Wan-Chun Huang	Respiratory Physician<br>MD MPH<br>PhD Candidate	Woolcock Institute of Medical Research<br>University of New South Wales

2. ROLES OF RESEARCH STAFF

Position description: Principal investigators

The Principal Investigator will be responsible for the following:

- Development of the final study protocol, in consultation with all relevant parties
- Provide written (or email) approval of all forms and manuals of procedures, prior to their use
- Obtain relevant ethical approval from the University of Sydney Human Research Ethics Committee
- Conduct periodic monitoring visits to evaluate data integrity and compliance with protocols and regulations
- Oversee the data analysis of the study, and preparation of data for reporting
- Approve budgets that have been developed by project staff.

Position description: Country Director (Woolcock Institute, Hanoi)

- o Oversee the Project Coordinator in implementing the project in Vietnam, including weekly meetings (in person or by teleconference).
- o Approve budgets prepared by the Project Coordinator, according to office financial policies.
- o Approve all forms and manuals of procedures, prior to submission to the Principal Investigator for final approval
- o Support the Project Coordinator in liaising with Vietnamese government partners, and its affiliated health care facilities to implement the project
- o Ensure the project complies with all relevant legal and ethical guidelines

Position description: Project Coordinator (Woolcock Institute, Hanoi)

The Project Coordinator will fulfill the following key roles:

- Work collaboratively with the staff and leaders within Government health facilities to implement the project in accordance with the Manual of Procedures.
- Conduct training of local staff as required.
- Oversee data collection and entry, ensuring that data is entered electronically in an accurate and timely way.
- Ensure that any adverse events or activities undertaken in the Project that could harm the reputation of the research organizations, sponsors or any individuals are clearly documented and promptly reported to the Country Director.
- Maintain the equipment for the study, and ensure it is properly accounted for according to institutional policies.

Position description: Project Officer (Research student)

The Project Officer (Research student) will fulfill the following roles:

- Work with the Principal Investigator to develop a manual of procedures, forms, data management processes, monitoring and evaluation processes, training materials and participant information
- Support training of Vietnamese staff at District Clinics
- Prepare monthly progress reports for the Principal Investigator
- Work with the other Project Officer and the Project Coordinator to obtain local approvals
- Monitor data collection, and perform regular quality assurance and data checking
- Work with the other Project Officer to conduct regular monitoring and evaluation in each District Clinic Develop a report of 'lessons learned' from this study that will be applied to the VCAPS randomised controlled trial

Position description: Project Officer (Woolcock Vietnam)

The Project Officer (Woolcock) will fulfill the following roles:

- Support training of Vietnamese staff at District Clinics
- Prepare monthly progress reports for the Principal Investigator
- Work with the other Project Officer and the Project Coordinator to obtain local approvals
- Work with the other Project Officer to conduct regular monitoring and evaluation in each District Clinic
- Perform in-service training for District Clinic staff, in consultation with the Project Officer (Research student).

Position description: District Coordinator

In each District Clinic, a District Coordinator will be appointed to oversee the study locally. This person will be responsible for the following:

- Participating in training workshop
- Recruiting participants in the District
- Obtaining support from other District staff
- Recording summary data in a Register Book, for review each month
- Informing the Project Coordinator of Adverse Events / Protocol deviations in the District

Position description: District health workers

District Health workers involved in the care of patients with either chronic respiratory symptoms or patients who are current smokers will:

- Participate in training and accreditation for the study
- Recruit study participants meeting the inclusion criteria
- Instruct patients to perform spirometry / peak flow

- Complete information in the study Register Book and other relevant forms
- Collect the contact details of smokers, to allow follow-up after discharge
- If a current smoker themselves, and they agree to participate, the health workers will be invited to participate in a health worker smoking cessation program

Position Description: Provincial Health workers

- Participate in training
- Conduct monitoring visits, with the Research Officers
- Provide advice about the suitability of the Manual of Procedures and other study documents, during the localisation process.

3. BACKGROUND AND RATIONALE

Chronic respiratory disease (CRD), one of the four major noncommunicable diseases worldwide, consists of a variety of diseases such as chronic obstructive pulmonary disease (COPD) and asthma. COPD is predicted to become the third biggest cause of death by 2030. Asthma is also one of the most important causes of disability worldwide. Both conditions are characterized by chronic airway inflammation and exacerbations that are recognised by sudden worsening of pulmonary function and respiratory symptoms. They also share risk factors, such as smoking, air pollution and respiratory infections.

In Vietnam, both COPD and asthma are common health issues that pose significant burden to the health system. COPD was estimated to affect 7.1% of adult in a northern population[1]. The prevalence of asthma was shown to be 5.6% in adult and 13.9% in school children[2,3]. Based on a review on noncommunicable disease by Vietnam Ministry of Health, COPD and asthma account for more than 7% of total disability adjusted life years across the country[4].

Evidence from previous studies have demonstrated cost-effective interventions to prevent and reduce the burden of CRD. Interventions on tobacco control are also effective in reducing symptoms, preventing disease deterioration and exacerbations. However, despite available evidence-based international guidelines and cost-effective interventions, barriers lie in between those approaches and vulnerable populations, particularly in resource-limited settings[5-7].

Challenges are there to implement optimal care in Vietnam. Recent surveys in Vietnam showed low percentage of asthma guideline adherence among primary care physicians and insufficient awareness of hospital doctors toward COPD management[8,9]. Among patients with COPD, less than 10% received any form of inhaled medications and 24% had required an unplanned health care visit. The availability and affordability of effective treatment are also a barrier that may hinder proper patient management[10,11]. Furthermore, national policy to reduce tobacco smoking, one major risk factor of CRD, has not yet been successful, with 45% of males still smoking in 2015[12]. The gap between evidence-based practice and current disease management requires further investigation to address the burden of CRD.

In light of this, two baseline surveys, which kick off the Vietnam COPD, Asthma and

Prevention of Smoking (VCAPS) study, are currently underway to elucidate the current context and barriers to optimal care. The VCAPS 1 will contribute to an understanding of respiratory disease and smoking practices among patients presenting to health facilities. The current practices and attitudes regarding disease management and tobacco control will be characterised in the VCAPS 2 study.

The present study (VCAPS 3) will serve as a pilot study for a randomised controlled trial (VCAPS 4, a cluster RCT) that aims to show the effectiveness of an integrated public health intervention to reduce the number of exacerbations among patients with symptomatic CRD. The results of the two surveys and this pilot study will inform the design of the VCAPS 4 RCT.

4. OBJECTIVES

The project proposed in this document (VCAPS 3) is a prospective cohort study that will inform the design of the VCAPS RCT (the VCAPS 4 RCT). The aim of the VCAPS 4 RCT will be to demonstrate the effectiveness of an integrated public sector intervention to reduce the number of exacerbations among individuals with CRD presenting to healthcare facilities. The specific objectives of the VCAPS 3 study (the present study) are as the following.

4.1. Chronic respiratory disease - objectives

(a) To determine the proportion of participants with at least one exacerbation of respiratory symptoms during a 12 month period, with treatment with inhaled corticosteroid/long acting beta agonist (ICS/LABA) therapy, according to a stepped symptom-based algorithm.

(b) To evaluate progression through the 'cascade of care' in the management of CRD. Steps in the cascade will include:

- The proportion of patients attending a health facility presenting with chronic respiratory symptoms consistent with CRD.
- The proportion of patients with chronic respiratory symptoms who initiate diagnostic assessment
- The proportion of patients who complete spirometry / peak expiratory flow.
- The proportion of patients completing diagnostic assessment who are diagnosed with CRD.
- The proportion of patients with CRD who commence treatment with ICS/LABA therapy, according to the study algorithm.
- The proportion of patients who are attend re-assessment 4 weeks after initiation of therapy
- The proportion of patients continuing recommended treatment 12 months after their initial presentation

(c) To evaluate the feasibility and acceptability of a stepped algorithm using ICS/LABA therapy for the management of CRD

4.2. Smoking cessation for patients - objectives

(a) To determine the proportion of patients enrolled in the smoking cessation programme (SCP) who achieve validated smoking abstinence for at least the previous 30 days. 12 months after enrolment

(b) To evaluate progression through the ‘cascade of care’ for the management of prevalent smoking among all patients presenting to the health facility. Steps in the cascade will include:

- The proportion of smokers among those presenting to health facilities
- The proportion of current smokers given brief advice
- The proportion of smokers enrolled in a smoking cessation program (SCP)
- The proportion of those enrolled in the SCP who complete initial outpatient counseling and receive smoking cessation material
- The proportion of those enrolled in the SCP who report making at least one quit attempt, lasting at least 30 days, during the 12-month follow-up period
- The proportion of smokers who report being abstinent from smoking for at in the prior 30 days at 3, 6, and 9 months after recruitment

(c) To evaluate the acceptability of the smoking cessation intervention among patients 3 months after enrolment, using both quantitative and qualitative methods

4.3. Smoking cessation for healthcare workers - objectives

(a) To determine the proportion of HCWs enrolled in the SCP who achieve validated smoking abstinence for at least 30 days 12 months after enrolment

(b) To determine the proportion of healthcare workers (HCWs) who participate in a staff smoking cessation training program

(c) To evaluate progression through the ‘cascade of care’ for the management of prevalent smoking among HCWs who are current smokers. Steps in the cascade will include:

- To determine the proportion of HCWs who are current smokers who participate in the SCP
- The proportion of enrolled smoking HCWs who make at least one quit attempt, lasting at least 30 days, during the 12-month follow-up period
- To determine the proportion of enrolled smoking HCWs who remain abstinent from smoking for at least 30 days at 3, 6, and 9 months after enrolment in the

SCP

(e) To evaluate the acceptability of the intervention for healthcare providers, using both quantitative and qualitative methods.

5. OVERVIEW OF STUDY DESIGN

This is a prospective cohort study aims to assess the feasibility of an integrated public sector intervention that is hypothesised to (a) reduce the number of exacerbations among individuals with CRD presenting to healthcare facilities and (b) reduce the proportion of current smokers among patients presenting to healthcare facilities and HCWs in the facilities.

Three interventions will be implemented, including (a) chronic respiratory disease intervention, (b) patient smoking cessation intervention, and (c) health facility smoking cessation intervention. Patients with CRD, diagnosed by the demonstration of airflow limitation or a respiratory symptom questionnaire, will be recruited to the chronic respiratory disease intervention. Patients who are current smokers will be invited to join the patient smoking cessation intervention. The health facility smoking cessation intervention is a facility-wide program that will implement a 'smoke free hospital' approach, including smoking cessation advice and facility-wide interventions to mitigate smoking among patients and staff in the selected district facilities. Training will be provided to HCWs in the three district facilities before participant recruitment.

The recruitment period for both components of the study will be 16 weeks. The follow-up period will be 12 months.

Co-primary outcomes

Co-primary outcomes of the study are:

1. Chronic respiratory disease intervention: proportion of patients with CRD who have at least one exacerbation during follow-up
2. Patient smoking cessation intervention: proportion of smoking abstinence at 12 months, among smoking patients enrolled in the program, and
3. Health facility smoking cessation intervention: proportion of HCWs with smoking abstinence at 12 months.

Definitions for each of these terms are included in the glossary.

The overview of the study is shown in Figure 1.

Figure 1. Overview of VCAPS3 Study

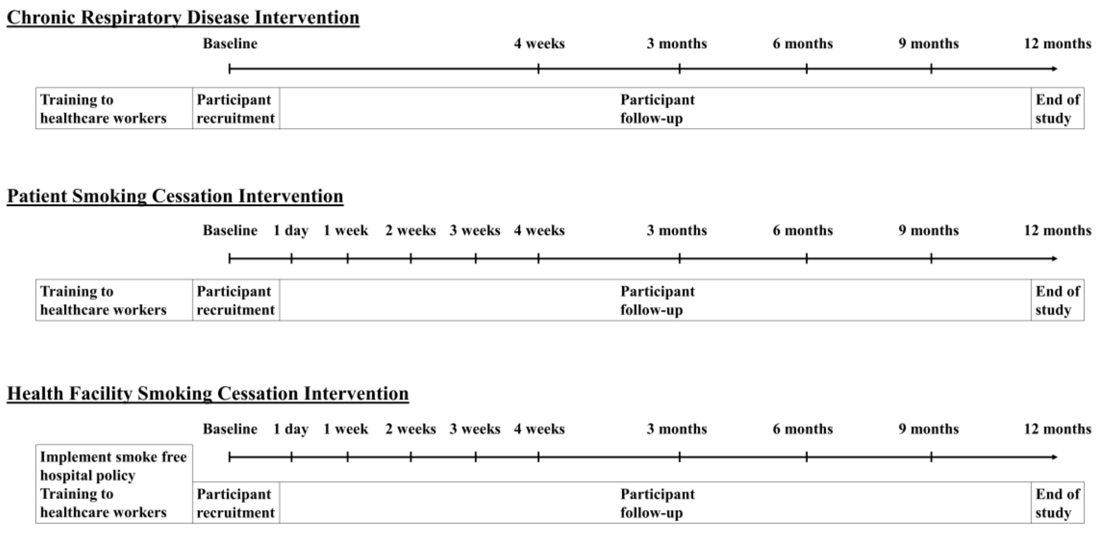

6. DETAILED PROCEDURES OF THE STUDY

Study design:

This is a prospective cohort study.

6.1. Settings

This study will be conducted in three government district health facilities in rural districts of Hanoi Capital.

6.2. Study population

The study population is patients presenting consecutively to selected district facilities and HCWs in the facilities. Specifically, patients who present to selected district facilities with chronic respiratory symptoms are considered eligible for the chronic respiratory disease intervention. Patients visiting the facilities who are current smokers are eligible for patient smoking cessation intervention. Health facility smoking cessation intervention will be a facility-wide intervention in which smoking employees of the facilities are invited for the SCP.

6.3. Inclusion criteria for chronic respiratory disease intervention

(a) Patients aged 12 years and over presenting to district health facilities, AND

(b) At least one of cough, dyspnea, wheeze, or chest tightness, AND

(c) A history of at least one prior episode of respiratory symptoms that has required attendance at a public or private health care facility or private pharmacy within the past two years, AND

(d) Demonstrated airflow limitation, defined as a pre-bronchodilator FEV1/FVC ratio less than 70% on spirometry or a peak flow meter reading less than 80% of predicted peak expiratory flow rate. Spirometry is the preferred method. Patients who cannot achieve acceptable spirometry result[13] will perform peak expiratory flow instead as performing peak expiratory flow is less complicated and safer than spirometry. The contraindications are listed in Table 1. OR

(e) Probable asthma: answering yes to at least three of the nine questions in the respiratory symptom questionnaire (Table 2). The questionnaire is adopted from a previously validated questionnaire[14].

(f) An alternative diagnosis, other than obstructive airway disease, is unlikely to explain the respiratory symptoms. Common alternative diagnoses and their features are listed in Table 3.

(g) Intends to be resident in Hanoi Capital for the next 12 months.

Table 1. Conditions that an acceptable lung function result cannot be obtained
**Absolute contraindications**
Within 1 month after acute myocardial infarction
Ascending aortic aneurysm
Acute pulmonary embolism
Active tuberculosis under treatment
**Relative contraindications**
Undiagnosed haemoptysis
Current pneumothorax
Thoracic/abdominal/brain/eye surgery in the last 3 months
Confused/demented patients
**Poor performance***
**Patient refusal**
*Does not meet acceptable blow criteria defined by American Thoracic Society/European Respiratory Society Task Force[13]

Table 2. Respiratory Symptom Questionnaire		
In the last 4 weeks, if you run, or climb stairs fast did you ever		
cough?	□ Yes	□ No
wheeze?	□ Yes	□ No
get tight in the chest?	□ Yes	□ No
In the last 4 weeks, was your sleep ever broken by		
wheeze?	□ Yes	□ No
difficulty with breathing?	□ Yes	□ No
In the last 4 weeks, did you ever wake up in the morning with		
wheeze?	□ Yes	□ No
difficulty with breathing?	□ Yes	□ No
In the last 4 weeks, did you ever wheeze		
if you were in a smoky room?	□ Yes	□ No
if you were in a very dusty place?	□ Yes	□ No

Table 3. Common alternative diagnoses		
**Diagnosis**	**History and physical examination**	**Radiology and lab**
Upper respiratory tract infection without exacerbation of COPD/asthma	History of rhinorrhoea, sore throat, cough and fever. Examination shows no wheeze on chest exam.	No Chest X-ray abnormalities
Heart failure	History of acute myocardial infarction, orthopnoea, paroxysmal nocturnal dyspnoea. Examination shows fine basal crackles, engorged jugular vein, lower limb oedema	Chest X-ray: cardiomegaly, perihilar hazeness, pulmonary edema, bilateral pleural effusion
Pneumonia	Purulent sputum with or without fever for days	Chest X-ray: consolidation; leukocytosis with neutrophilia
Tuberculosis	Progressive cough with sputm production for more than 3 weeks; may have fever	Chest X-ray: fibrotic change, consolidation and/or cavitation; Sputum positive for AFB or MTB PCR (GeneXpert)
Pneumothorax	Acute dyspnoea, pleuritic chest pain	Chest X-ray: a visceral pleural line without distal lung markings
Other respiratory complaint	History typical for other condition unrelated to airways disease.	

For patients visiting emergency department (ED) with (a), (b) and (c) criteria, assessment of airflow limitation will be done after initial treatment at ED. If for any reason that a patient cannot do spirometry and peak flow meter, the follow-up at outpatient department within 14 days will be arranged.

6.4. Inclusion criteria for patient smoking cessation intervention

(a) Patients at the health facility aged 12 years and over

(b) Has smoked 100 cigarettes in his or her lifetime

(c) Currently smokes cigarettes (defined as smoking in the previous month)[15]

(d) The patient agrees to participate the SCP

(e) The patient is able to communicate effectively with HCWs

(f) Intends to be resident in Hanoi Capital for the next 12 months.

6.5. Inclusion criteria for smoking cessation intervention for health workers

The health facility smoking cessation intervention is a facility-wide intervention. Therefore, all HCWs in the facilities will be eligible to be included in the intervention. HCWs who meet the following criteria are eligible for the SCP aiming to help them quit.

(a) Healthcare worker employed by the facility

(b) Has smoked 100 cigarettes in his or her lifetime and who currently smokes cigarettes (defined as smoking in the previous month) [15]

(c) The healthcare worker agrees to participate.

Accordingly, there will be four groups of study participants. Figure 2 summarises the inclusion algorithm.

Figure 2. Patient recruitment algorithm

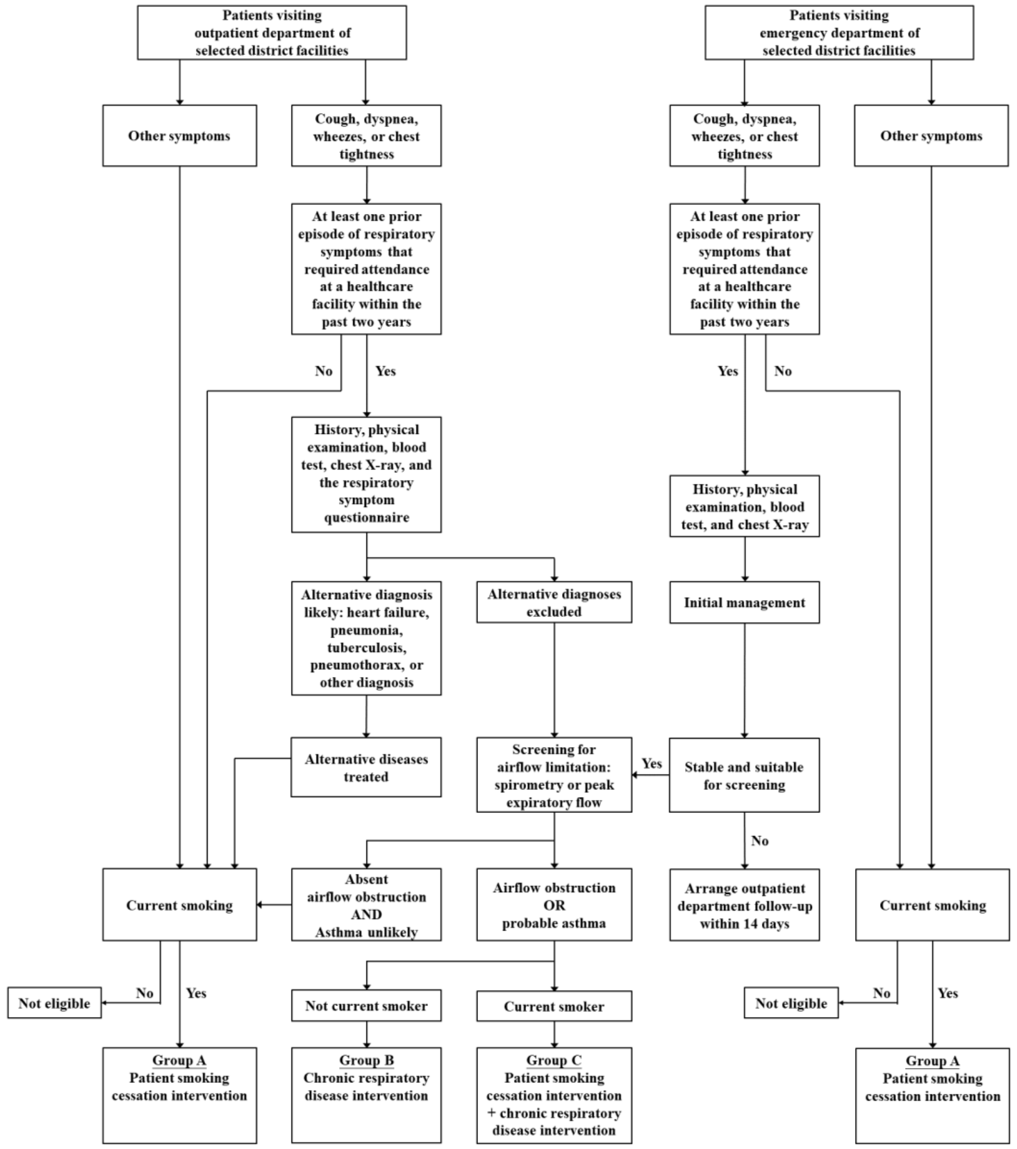

Group A: **Current smokers who do not have airflow limitation.** Patients in this group will be offered the patient smoking cessation intervention.

Group B: **Patients with airflow limitation who do not smoke.** Patients in this group will be offered the CRD intervention.

Group C: **Current smokers who have airflow limitation.** Patients in this group will be offered both the patient smoking cessation intervention and the CRD intervention.

Finally, Group D will be HCWs in the three district health facilities where the facility-wide smoking cessation program is conducted.

6.6. Exclusion criteria

(a) Patients who are unable to provide informed consent

(b) Patients who are allergic to budesonide or formoterol will be excluded from the chronic respiratory disease intervention.

(c) Women who know they are pregnant will be excluded from the chronic respiratory disease intervention.

6.7. Interventions and follow-up

Figure 3 summarises the procedures of the three interventions.

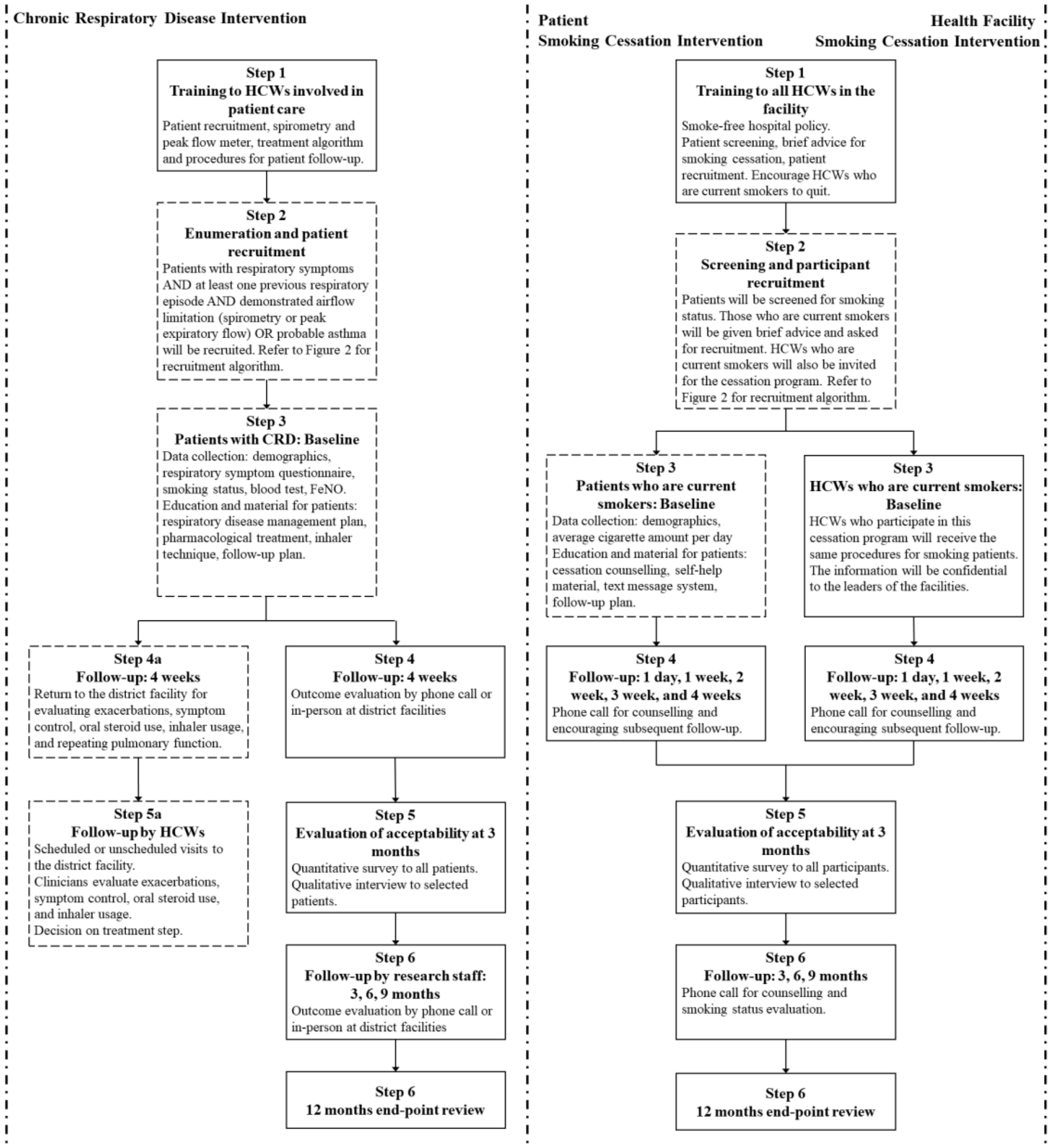

Figure 3. Flowchart of VCAPS3 Study*

***Note:** Steps in solid squares are done by research staff; steps in dotted squares are done by hospital staff.
Abbreviations: HCW, healthcare workers; CRD, chronic respiratory disease; mMRC, modified Medical Research Council; CAAT, Chronic Airways Assessment Test; FeNO, fractional exhaled nitric oxide

6.7.1. Healthcare worker training

Health workers at participating districts will be trained to implement:

- The chronic respiratory disease intervention
- The patient smoking cessation intervention
- The health facility smoking cessation intervention

The investigators will provide a training session regarding the three interventions before implementing them. The components included are listed in Table 4.

Table 4. Components of the training for healthcare workers

**Chronic respiratory disease intervention**	
	Diagnosis of chronic respiratory disease
	Patient recruitment
	Contraindications, instruction and interpretation of spirometry/peak expiratory flow
	Treatment algorithm
	Inhaler technique
	Follow-up procedures
**Patient smoking cessation intervention and health facility smoking cessation intervention**	
	Smoke-free hospital policy
	Patient screening
	Patient counselling
	Patient recruitment
	Participation of healthcare workers who are current smokers in the smoking cessation program
	Follow-up procedures

6.7.2. The chronic respiratory disease intervention

After excluding alternative causes of their respiratory symptoms, patients will be screened with spirometry or peak expiratory flow if spirometry cannot be performed (Table 1). Those who meet the inclusion criteria will be invited to participate in the chronic respiratory disease intervention. The chronic respiratory disease intervention will follow an algorithm that consists of the following elements:

1. Participant demographics, blood tests (full blood count including white blood cell count, eosinophil count), and fractional exhaled nitric oxide will be recorded by clinic staff in a record sheet designed for this study.
2. Pharmacological treatment: patients will be advised to use inhaled medicines, based on the pharmacological treatment algorithm (Figure 4), starting with step 1. Doctors in the facilities will evaluate the treatment step at each follow-up assessment visit (both scheduled or unscheduled). They will escalate treatment to a higher step if the participant has ongoing symptoms consistent with poor control, or had an acute exacerbation since last visit.

Figure 4. Pharmacological treatment algorithm

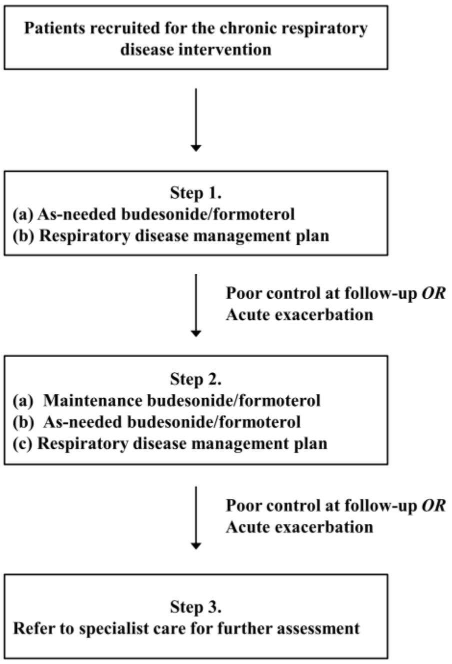

As-needed budesonide/formoterol: the patient is suggested to use budesonide/formoterol (160/4.5 µg/puff) to relieve day-to-day respiratory symptoms when it's necessary.

Maintenance budesonide/formoterol: the patient is suggested to use budesonide/formoterol (160/4.5 µg/puff) two times (two inhalations for each time, i.e. two inhalations in the morning and two inhalations in the evening) a day regularly.

Respiratory disease management plan: a written material given to all recruited patients that includes information about the diagnosis, disease management, symptom monitoring, treatment algorithm, inhaler technique, and smoking cessation

Poor control at follow-up: the patient will complete the symptom control questionnaire when returning to outpatient clinic for assessment. A score of less than 20 is considered poor control.

Acute exacerbation: an acute worsening of respiratory symptoms within days that does not respond to as-needed budesonide/formoterol and requires oral steroid or attendance to a hospital for acute treatment.

Refer to specialist care: patients whose respiratory symptoms cannot be controlled by maintenance plus as-needed budesonide/formoterol will be referred to a respiratory specialist at the outpatient department of Hanoi Lung Hospital.

3. All patients will be given a leaflet about CRD and a respiratory disease management plan at the time of enrolment, regardless of the severity of their symptoms. The respiratory disease management plan includes an exacerbation plan that explains the use of oral steroids and oral antibiotics (Table 5). When a patient has an acute worsening of respiratory symptoms that requires a total dose of more than 6 inhalations of as-needed budesonide/formoterol a day over a period of 3 days, prednisolone 1mg/kg (no more than 40mg) should be added for 3-5 days.
4. In addition, if a patient reports purulent sputum (i.e. yellow or green colour and increased volume compared to normal) +/- fever of over 37.5ºC, then an oral antibiotic (amoxicillin/clavulanic acid OR cefuroxime are suggested) should be offered.
5. Patients who meet the condition for using oral steroids +/- oral antibiotics should seek a medical review to obtain the medicines indicated in the exacerbation plan.

Table 5. Exacerbation plan for patients with chronic respiratory disease

**Oral steroids**	Condition to initiate	Acute worsening of respiratory symptoms that requires more than 6 doses of prn budesonide/formoterol a day over a period of 3 days
	Dosage of steroids	Prednisolone 1mg/kg (no more than 40mg) for 3-5 days
**Oral antibiotics**	Condition to initiate	Purulent sputum +/- fever that acompanies above symptoms
	Choice of antibiotics	Amoxicillin/clavulanate for 7-10 days<br>Alternative for patients allergic to penicillin: cefuroxime for 7-10 days

6. Pharmacists at district facilities will provide education on instructing patients to use inhaled medicines at the time they dispense the medication, where possible. Otherwise, another member of the health care workforce will deliver education about inhaler use.
7. Routine follow-up at clinics will be scheduled by staff at district facilities. At these visits, the treating health workers will discuss the following with patients:
    - symptom control
    - need to step up according to the algorithm
    - need for use of oral steroids (see Table 5)
    - assessment and treatment changes
8. Unscheduled follow-up will occur if patients have symptoms that are not adequately controlled despite use of the 'step up' algorithm. At these visits, trained health workers will discuss the following with patients:
    - symptom control
    - need to step up according to the algorithm
    - need for use of oral steroids (see Table 5)
9. Assessment and treatment changes will be documented by district staff in the record sheet.
10. For patients at step 3 of the treatment algorithm (Figure 4), specialist care is necessary. These patients will be referred to Hanoi Lung Hospital for further clinical assessment and further treatment suggestions by a respiratory specialist at outpatient department.
11. For women who become pregnant during the study, they will consult their doctor to decide whether to continue treatment.

Definitions of poor symptom control and acute exacerbation

12. Definition for poor symptom control: a symptom control questionnaire (Table 6) derived from Asthma Control Test will be used for symptom control evaluation. A score of less than 20 will be considered poor symptom control.
13. Definition for acute exacerbation: an acute worsening of respiratory symptoms within days that requires oral steroid, as described in the exacerbation plan, or a hospital visit.

4 week follow-up visit

14. Patients will attend the clinic 4 weeks after their enrolment.
15. At this 4 week visit, a follow-up spirometry will be performed (before and after bronchodilator [salbutamol]). This will allow an assessment of the degree of bronchodilator reversibility. Patients who cannot perform spirometry will have peak flow meter (Table 1).

Follow-up

16. Patients will be followed by research staff for outcome evaluation at 4 weeks, 3 months, 6 months, 9 months, and 12 months after discharge from a district facility. The following outcomes will be recorded in a register book:
    - symptom control questionnaire score (Table 6),
    - mMRC Dyspnea Scale (Table 7),
    - CAAT score (Table 8),
    - hospital visits due to exacerbations, and
    - Self-reported adherence to recommended frequency of dosing.

Table 6. Symptom control questionnaire

1. In the past 4 weeks, how much of the time did your respiratory symptoms (shortness of breath, wheezing, cough and/or chest tightness) keep you from getting as much done at work, school or at home?

□ All of the time (1) □ Most of the time (2) □ Some of the time (3) □ A little of the time (4) □ Not at all (5)

2. During the past 4 weeks, how often have you had shortness of breath?

□ More than once a day (1) □ Once a day (2) □ 3 to 6 times a week (3) □ Once or twice a week (4) □ Not at all (5)

3. During the past 4 weeks, how often did your respiratory symptoms (shortness of breath, wheezing, cough, and/or chest tightness) wake you up at night or earlier than usual in the morning?

□ 4 or more nights a week (1) □ 2 to 3 nights a week (2) □ one night a week (3) □ Less than one night a week (4) □ Not at all (5)

4. During the past 4 weeks, how often have you used your rescue inhaler?

□ 3 or more times a day (1) □ 1 or 2 times a day (2) □ 2 or 3 times a week (3) □ Once a week or less (4) □ Not at all (5)

5. How would you rate your breathing problem control during the past 4 weeks?

□ Not controlled (1) □ Poorly controlled (2) □ Somewhat controlled (3) □ Well controlled (4) □ Completely controlled (5)

Total score:

A score of less than 20 is considered poor control.

Table 7. modified Medical Research Council Dyspnoea Scale

Please ask the patient to choose one from the five options

[ ]	0	I only get dyspnea with strenuous exercise
[ ]	1	I get dyspnea when hurrying on level ground or walking up a slight hill
[ ]	2	I walk slower than people of the same age because of dyspnea or have to stop for breath when walking at my own pace
[ ]	3	I stop for breath after walking about 100 m(91 m) or after a few minutes on level ground
[ ]	4	I am too dyspneic to leave house or I am breathless when dressing

Table 8. Chronic Airways Assessment Test

Please ask the patient to indicate his/her status (on a scale of 0 to 5) for each of the 8 questions.

I never cough	[0] [1] [2] [3] [4] [5]	I cough all the time
I have no phlegm (mucus) in my chest at all	[0] [1] [2] [3] [4] [5]	My chest is full of phlegm (mucus)
My chest does not feel tight at all	[0] [1] [2] [3] [4] [5]	My chest feels very tight
When I walk up a hill or one flight of stairs I am not breathless	[0] [1] [2] [3] [4] [5]	When I walk up a hill or one flight of stairs I am very breathless
I am not limited doing any activities at home	[0] [1] [2] [3] [4] [5]	I am very limited doing activities at home
I am confident leaving my home despite my lung condition	[0] [1] [2] [3] [4] [5]	I am not at all confident leaving my home because of my lung condition
I sleep soundly	[0] [1] [2] [3] [4] [5]	I don't sleep soundly because of my lung condition
I have lots of energy	[0] [1] [2] [3] [4] [5]	I have no energy at all

Total score:___________

6.7.3. The patient smoking cessation intervention and the health facility smoking cessation intervention

This study will apply four strategies to reduce smoking within the health facility and enhance patient smoking cessation.

1. **Facility-wide strategy:** district facilities will implement and enforce existing smoke-free hospital procedures (based on legal documents from Ministry of Health and a practical guide developed by the Vietnam Committee on Smoking and Health).

2. **Delivery of brief smoking cessation advice by staff** : HCWs will be trained to screen patients for smoking status, as indicated in Figure 1 and Figure 2, and offer brief counselling to patients with the 5As approach (Table 9)[17].

Table 9. 5As approach of brief smoking cessation counselling	
**Ask**	Ask and record smoking status
**Advice**	Advice on the benefits of quitting
**Assess**	Assess motivation to quit
**Assist**	Aid the patient in quitting
**Arrange**	Schedule follow-up contact

3. **Smoking cessation materials:**
   - Written material to help patients quit will also be offered to all patients
4. **Integrated phone call and interactive two-way text message follow-up:**
   - See following section and Appendix 1 for the procedures.

Enrolment of smokers

- All patients attending the clinic will be asked about their current smoking status and will be documented.
- The proportion of patients receiving screening and counselling for smoking will be reported.
- Patients who agree to participate and provide consent will receive self-help material.

Follow-up of smokers

- Recruited patients with receive a phone call from research staff to provide smoking cessation counselling within 24 hours.
- They will then receive outpatient phone follow-up from a centralised call centre, by trained health workers, to support their cessation at:
  - 1 week, 2 weeks, 3 weeks, 4 weeks, 3 months, 6 months, 9 months, and 12 months.
  - Phone call follow-up will be integrated with an interactive two-way text message service (see Appendix 1 for examples of the text messages)

- Urinary cotinine will be used for biochemical verification of smoking cessation at 12 months[19].

Staff smoking cessation

- Staff who are identified as current smokers will be invited to participate. They will receive the same follow-up, materials, and services indicated above. Staff counseling will be confidential, with the leadership of the district clinics not able to access the records of health workers involved in this program.

6.8. Outcomes

The findings of this study will be used to inform the implementation of the VCAPS RCT (the VCAPS 4 study). We will measure a number of clinical and process indicators for the three interventions.

6.8.1. Primary outcome of the chronic respiratory disease intervention: The proportion of patients presenting with respiratory symptoms who have airflow limitation or probable asthma who have at least one exacerbation during the 12 months following enrolment

At each follow-up for outcome evaluation (4 weeks, 3, 6, and 9 months) and the 12 month end-point review, the research staff will ask about acute exacerbations that will be used for calculating the proportion.

6.8.2. Secondary outcome of the CRD intervention:

The proportion of patients completing the 'cascade of care' for CRD management. The cascade of care refers to each step in the diagnostic and treatment pathway that is expected according to optimal clinical practice. This study will measure the progression through each of the following steps:

1. **The proportion of patients presenting with chronic respiratory symptoms consistent with CRD**

   Among patients presenting with respiratory symptoms, how many of them have at least one prior episode of respiratory symptoms that has required

attendance at a public or private health care facility or private pharmacy within the past two years OR score three or more in the respiratory symptom questionnaire.

2. **The proportion of patients with chronic respiratory symptoms who initiate diagnostic assessment in health care facilities**
   How many patients among those in the previous step begin diagnostic evaluation, including spirometry (or peak flow meter if the patient cannot or is contraindicated to perform spirometry).
3. **The proportion of patients who complete diagnostic assessment**
   How many patients among those in the previous step successfully complete a clinical assessment, and spirometry OR peak expiratory flow OR documented ineligibility for both spirometry and peak expiratory flow in the record sheet.
4. **The proportion of patients completing assessment who are diagnosed with CRD**
5. **The proportion of patients with CRD who commence appropriate treatment, as defined by the study algorithm (see Figure 3)**
6. **The proportion of patients who are attend a clinical assessment 4 weeks after initiation of therapy** (includes up to a maximum period of 8 weeks after enrolment).
   Among patients prescribed with prn budesonide/formoterol, how many (a) return to the district facility to attend scheduled clinical evaluation at 4 weeks, and (b) have completed either spirometry OR peak flow meter OR are ineligible for spirometry at that visit.
7. **The proportion of patients continuing recommended treatment 12 months after their initial presentation**
   Among patients who commence therapy, how many of them inform research staff that they continue to take the recommended therapy algorithm until the end-point review.

6.8.3. Primary outcome of the patient smoking cessation intervention: biochemically verified self-reported abstinence at 12 months[19,20]

Among enrolled current smokers who undergo the patient smoking cessation intervention the proportion of participants who
(a) reported abstinence from smoking for at least 30 days AND
(b) had a negative urinary cotinine result.
This outcome will be assessed any time from week 51 through to week 60 after enrolment.

6.8.4. Secondary outcomes of the patient smoking cessation intervention

6.8.4.1. The proportion of patients completing the 'cascade of care' for the management of smoking

1. **The proportion of current smokers among patients presenting to health facilities**
   Among patients presenting to selected health facilities, how many of them are current smokers.
2. **The proportion of current smokers with documentated brief counselling by health workers**
   Among current smokers, how many have the health workers documented they have provided brief advice.
3. **The proportion of current smokers agreeing to enrol in the SCP**
   Among patients receiving counselling from the health workers, how many agree to enrol in the smoking cessation intervention
4. **The proportion of current smokers who enrolled in the SCP who complete initial outpatient counseling and receive smoking cessation material**
5. **The proportion of those enrolled in the smoking cessation program who report at least one quit attempt that lasts at least 30 days during the 12 month follow-up period.**
6. The proportion of those enrolled in the SCP who remain abstinent from smoking for at least 30 days at 3, 6, and 9 months

6.8.4.2. Self-reported 7-day point prevalence of smoking abstinence[20]

The proportion of current smokers participating in the smoking cessation program

who report no smoking in past 7 days at 4 weeks, 3, 6, 9 and 12 months.

Abstinence from smoking: health-care workers

The outcome measures described in 6.8.3 and 6.8.4 will also be measured for HCWs, and reported separately. Follow-up will be at the same time periods as for the patients.

6.8.5. Secondary outcomes shared by the interventions: Attitude and perceptions of patients and HCWs regarding the interventions

All participants will be asked to complete a quantitative survey at 3 months (either in-person or by phone call interview by research staff).

6.8.6. Secondary outcomes shared by the interventions: Qualitative interviews

A qualitative assessment will also be conducted at 3 months for a sample of up to 20 patients and healthcare providers receiving each of the interventions (i.e. a total of up to 20 interviews). A purposive sampling approach will be applied. The focus will be how to improve acceptance and scalability of the interventions.

7. GLOSSARY

7.1. Definition of key terms

Term	Definition
Acute exacerbation	An acute worsening of respiratory symptoms within days that requires systematic corticosteroid or management at a healthcare facility (commune health centre, clinic, or hospital)
Biochemically verified abstinence	Smoking abstinence with a negative urinary cotinine result
Chronic respiratory disease	A diagnosis made by (a) demonstration of airflow limitation (either by spirometry [FEV1/FVC < 70%] or peak flow meter [< 80% of predicted peak expiratory flow rate]) OR (b) score three or more in the respiratory symptom questionnaire.
Current smoker	An individual who has smoked 100 cigarettes in his or her lifetime and has smoked in the previous month
Healthcare workers	Employees in health facilities with a health-related profession
Private healthcare facility	A healthcare facility that is not owned by the Vietnamese government
Private pharmacy	A pharmacy that is privately owned and is not a department within another healthcare facility
Public healthcare facility	A healthcare facility that is owned by the Vietnamese government
Self-reported 7-day smoking abstinence	A self-report of no smoking within the last 7 days
Self-reported abstinence	A self-report of no smoking in the last 30 days

7.2. Abbreviations

**Abbreviation**	**Term**
COPD	Chronic obstructive pulmonary disease
CRD	Chronic respiratory disease
CAAT	Chronic Airways Assessment Test
ED	Emergency department
FeNO	Fractional exhaled nitric oxide
FEV1	Forced expiratory volume in 1 second
FVC	Forced vital capacity
HCWs	Healthcare workers
mMRC	Modified Medical Research Council
ppm	Parts per million
RCT	Randomised controlled trial
SCP	Smoking cessation program
VCAPS	Vietnam COPD, Asthma and Prevention of Smoking

8. SAMPLE SIZE

The following table summarises the expected number of patients who will present to health facilities during the 16-week recruitment period.

**District health facility name**	**Expected number of patients enrolled for CRD intervention**	**Expected number of patients enrolled for smoking cessation intervention**	**Expected number of HCWs who smoke at enrolment and agree to participate in smoking cessation intervention**
Thach That	100	130	30
Me Linh	100	130	30
Thuong Tin	100	130	30
**Total**	**300**	**390**	**90**

9. ANALYTIC METHODS

In this study, we will calculate the proportion of patients participating in CRD intervention who have at least one exacerbation and proportion of participants joining smoking cessation intervention who quit smoking during the 12-month follow-up period. The proportions of population participating and following the procedures along the study process, namely the cascade of care, will also be obtained. Inferential statistics will be used to identify risk factors for drop out during the cascade of care.

The results will be compared with data from existing literature. Reasons for drop-out and nonadherence will be analysed.

The abovementioned analysis, along with the qualitative assessment of attitude and perceptions regarding the two interventions, will be used to guide the design of the VCAPS 4 study.

10. KNOWLEDGE DISSEMINATION PLAN

The findings of this pilot study and the main study will be published in a peer reviewed international scientific journal. The findings will be provided in a Policy Brief to the Vietnam Ministry of Health, to contribute to future policy development. The outcomes will also be presented at the annual scientific meeting of the Global Alliance for Chronic Diseases, in order to inform the application of this intervention in other low- and middle-income countries.

11. TIMELINE

**Jan 2019**	Complete proposal and data collection tool
**Jan 2019 –Feb 2019**	IRB application<br>Develop manual of procedures
**Mar 2019**	Conduct training in three district health facilities<br>Pilot questionnaires and data collection tools
**Mar 2019 – Jun 2019**	Recruitment
**Mar 2019 – Aug 2020**	Follow-up and completion of data checking
**Sep 2020**	Data analysis completed
**Dec 2020**	Findings submitted for publication

12. APPENDIX

12.1. Appendix 1: Examples of text messages for smoking cessation intervention

The detailed procedure of text message service will be constructed based on literature[23,24] and be adopted to fit culture and local context in Vietnam. Listed here are some examples.

**Timing of text message**	**Content**
On the designated quit date	Today is the date! Today is the start of being quit forever. You can achieve it and we are here to help!
In response to text “crave”	We know it’s difficult. However, cravings generally last less than 5 minutes. Let’s try something to distract yourself! How about try sipping a drink slowly until the craving is over?
In response to text “lapse”	Don’t feel bad or guilty if you’ve smoked again. It is a normal part of the quitting process. Encourage yourself as you already stopped for a while. Keep going, don’t give up!
In response to text “call”	It’s OK to ask for help. Our staff will call you as soon as possible to help you!

13. REFERENCES

1. Lâm HT, Ekerljung L, Tu·ò·ng NV, Rönmark E, Larsson K, Lundbäck B. Prevalence of COPD by Disease Severity in Men and Women in Northern Vietnam. *COPD: Journal of Chronic Obstructive Pulmonary Disease* 2014; **11**(5): 575-81.

2. Lâm HT, Rönmark E, Văn Tường N, Ekerljung L, Kim Chúc NT, Lundbäck B. Increase in asthma and a high prevalence of bronchitis: Results from a population study among adults in urban and rural Vietnam. *Respiratory Medicine* 2011; **105**(2): 177-85.

3. Nga NN, Chai SK, Bihn TT, et al. ISAAC-based asthma and atopic symptoms among Ha Noi school children. *Pediatric allergy and immunology : official publication of the European Society of Pediatric Allergy and Immunology* 2003; **14**(4): 272-9.

4. Vietnam Ministry of Health, Health Partnership Group. Joined annual health review: Strengthening prevention and control of non-communicable disease. Hanoi: Vietnam Ministry of Health. 2014.

5. Beran D, Zar HJ, Perrin C, Menezes AM, Burney P. Burden of asthma and chronic obstructive pulmonary disease and access to essential medicines in low-income and middle-income countries. *The Lancet Respiratory medicine* 2015; **3**(2): 159-70.

6. Mortimer K, Cuevas L, Squire B, Thomson R, Tolhurst R. Improving access to effective care for people with chronic respiratory symptoms in low and middle income countries. *BMC proceedings* 2015; **9**(Suppl 10): S3.

7. van Gemert FA, Kirenga BJ, Gebremariam TH, Nyale G, de Jong C, van der Molen T. The complications of treating chronic obstructive pulmonary disease in low income countries of sub-Saharan Africa. *Expert Review of Respiratory Medicine* 2018; **12**(3): 227-37.

8. Nguyen VN, Nguyen QN, Le An P, Chavannes NH. Implementation of GINA guidelines in asthma management by primary care physicians in Vietnam. *International journal of general medicine* 2017; **10**: 347-55.

9. Chu Thi H, Phan Thu P, Vu Van G, Ngo Quy C. Late-breaking abstract: Knowledge, attitudes and practice of medical doctors in diagnosis and

management of COPD patients in Vietnam. *European Respiratory Journal* 2014; **44**(Suppl 58).

10. Nguyen A, Knight R, Mant A, Cao Q, Auton M. Medicine prices, availability, and affordability in Vietnam. *Southern Med Review* 2009; **2**(2): 2-9.

11. Nguyen TA, Knight R, Mant A, et al. Inflated medicine prices in Vietnam: a qualitative study. *Health Policy Plan* 2017; **32**(5): 647-56.

12. World Health Organization. Global Adult Tobacco Survey http://www.who.int/tobacco/surveillance/survey/gats/en/ (accessed July 19 2018).

13. Miller MR, Hankinson J, Brusasco V, et al. Standardisation of spirometry. *The European respiratory journal* 2005; **26**(2): 319-38.

14. Venables KM, Farrer N, Sharp L, Graneek BJ, Newman Taylor AJ. Respiratory symptoms questionnaire for asthma epidemiology: validity and reproducibility. *Thorax* 1993; **48**(3): 214-9.

15. Ryan H, Trosclair A, Gfroerer J. Adult Current Smoking: Differences in Definitions and Prevalence Estimates—NHIS and NSDUH, 2008; 2012.

16. Schatz M, Kosinski M, Yarlas AS, Hanlon J, Watson ME, Jhingran P. The minimally important difference of the Asthma Control Test. *The Journal of allergy and clinical immunology* 2009; **124**(4): 719-23.e1.

17. NSW Health. Managing Nicotine Dependence: A guide for NSW Health Staff. Sydney, Australia, 2015.

18. Department of Health. Local stop smoking services: key updates to the 2011/12 service and monitoring guidance for 2012/13. London: DH, 2012. Available at: www.wp.dh.gov.uk/publications/files/2012/09/9193-TSO-2900254-NHS-Stop-Smoking_Accessible.pdf.

19. West R, Hajek P, Stead L, Stapleton J. Outcome criteria in smoking cessation trials: proposal for a common standard. *Addiction (Abingdon, England)* 2005; **100**(3): 299-303.

20. Hughes JR, Keely JP, Niaura RS, Ossip-Klein DJ, Richmond RL, Swan GE. Measures of abstinence in clinical trials: issues and recommendations. *Nicotine &*

Tobacco Research 2003; **5**(1): 13-25.
21. Brose LS, Tombor I, Shahab L, West R. The effect of reducing the threshold for carbon monoxide validation of smoking abstinence--evidence from the English Stop Smoking Services. *Addictive behaviors* 2013; **38**(10): 2529-31.
22. Wee LH, West R, Mariapun J, et al. Should the threshold for expired-air carbon monoxide concentration as a means of verifying self-reported smoking abstinence be reduced in clinical treatment programmes? Evidence from a Malaysian smokers' clinic. *Addictive behaviors* 2015; **47**: 74-9.
23. Scott-Sheldon LA, Lantini R, Jennings EG, et al. Text Messaging-Based Interventions for Smoking Cessation: A Systematic Review and Meta-Analysis. *JMIR mHealth and uHealth* 2016; **4**(2): e49.
24. Free C, Knight R, Robertson S, et al. Smoking cessation support delivered via mobile phone text messaging (txt2stop): a single-blind, randomised trial. *Lancet (London, England)* 2011; **378**(9785): 49-55.
